# Dictionnaire De Médecine, Ou Répertoire Général Des Sciences Médicales Considérées Sous Les Rapports Théorique Et Pratique

**Published:** 1842-10-22

**Authors:** 


*Dictionnaire de Médecine, ou Répertoire Général des Sciences Médicales considérées sous les rapports théorique et pratique;* t. xxv., Paris, 1842, 8vo.

*Dictionary of Medicine*, &c. Vol. xxv. Paris, 1842, 8vo.

The twenty-fifth volume of the Dictionary contains a number of very important articles. Several of them, as Pleurisy, Pneumonia, Pneumothorax, are by M. Chomel, who treats these subjects with characteristic clearness and exactness. We notice that M. Chomel divides acute pleurisy into partial and general, comprising under the first head, costo-pulmonary, diaphragmatic, mediastinal, and interlobular pleurisy. Of these varieties, often so difficult to distinguish the diagnostic marks are lucidly pointed out. M. Chomel's remarks upon Läennec's hemorrhagic pleurisy are worth quoting. He says:

"Läennec has noticed, and described as a distinct variety of pleurisy, that which terminates in an effusion of blood, or bloody serosity into the pleura; he thinks that in these cavities exclusively are formed those thick, false-membranes of fibro-cartilaginous consistency, which, according to him, are composed of albumen and fibrine, and which cannot take place except in an effusion of this character. But this opinion is not in accordance either with autopsical results, which show that a purely serous liquid is often surrounded by fibro-cartilaginous membranes, or with the most recent chemical researches, which have detected fibrine in the ordinary false membranes, heretofore considered as purely albuminous. Again, the greater rapidity pointed out by Läennec as occurring in the formation of the effusion in question, and the increased intensity of pain insisted on by Broussais, are *not* of constant occurrence in hemorrhagic pleurisy. It, therefore, constitutes merely an anatomical variety of the disease, which cannot be specially recognized during life.

"I am more disposed," adds M. Chomel, "to admit a variety of pleurisy, which I would call the purulent variety. This may sometimes be recognized by the discharge of pus either through the bronchiæ, or an opening in the parietes of the thorax. In those cases in which the pus finds no external exit, this species of pleurisy may be suspected from the increased intensity of the general symptoms, particularly of the fever, especially when the extent of the pleurisy and the quantity of liquid thrown out are not in proportion to the violence of the symptoms."

The article Pneumonia is able and elaborate, but presents no points deserving special notice. The article Pneumothorax is by the same author. M. Chomel adopts as the explanation of the phenomenon of metallic tinkling, the passage of air through the liquid, which stops up the fistulous orifice communicating between the lung and pleura.

There are two lengthy articles on Surgery in this volume. The first is Affections of the Wrist, (Poignet) by M. Velpeau, who discusses many important points, connected with the subject. M. Velpeau finds no case recorded of luxation of the mass of bones of the carpus upon the fore-arm, unless there have been ruptures of bony ridges, tendinous grooves, or some fracture. He does not, therefore, admit simple luxations of the wrist. On the subject of fractures of lower extremity of the radius, M. Velpeau adopts the opinion recently put forth by Mr. Voillemier, that the superior fragment penetrates the inferior, which offers a larger surface and less resistance. He admits, however, besides, the transverse fracture, (described by Dr. J. Rhea Barton,) and an oblique fracture. For the treatment of fracture of the lower extremity of the radius, M. Velpeau proposes an immoveable apparatus.

There is an excellent article by M. Marjolin on the Surgical diseases of the breast, (Poitrine.) The subject of lead, (Plomb) is treated by MM. Orfila, Cazenave, and Soubeiran. From the cursory notice which we give of the contents of this volume, it will be seen that there is no flagging in the spirit and ability which have heretofore distinguished the work. We again recommend such of our readers as are not subscribers to it, to become so.

